# CBD, a precursor of THC in e-cigarettes

**DOI:** 10.1038/s41598-021-88389-z

**Published:** 2021-04-26

**Authors:** Zsuzsanna Czégény, Gréta Nagy, Bence Babinszki, Ákos Bajtel, Zoltán Sebestyén, Tivadar Kiss, Boglárka Csupor-Löffler, Barbara Tóth, Dezső Csupor

**Affiliations:** 1 Institute of Materials and Environmental Chemistry, Research Centre for Natural Sciences, Eötvös Loránd Research Network, Magyar tudósok körútja 2, Budapest, 1117 Hungary; 2 Department of Pharmacognosy, Faculty of Pharmacy, University of Szeged, Eötvös u. 6, Szeged, 6720 Hungary; 3 Institute for Translational Medicine, Medical School, University of Pécs, Szigeti út 12, Pécs, 7624 Hungary

**Keywords:** Natural products, Mass spectrometry, Risk factors

## Abstract

The use of cannabidiol (CBD) in electronic cigarettes is widespread. Previously, it was reported that CBD is partly transformed to THC in case smoking as a cigarette, however, the pyrolysis of this compound has not been assessed extensively. The aim of our study was to investigate the effect of temperature on the composition of pyrolysis products of CBD. The experiments were performed in the typical operating temperature range of e-cigarettes (250–400 °C) and at 500 °C under both inert and oxidative conditions, and the pyrolysis products were identified and quantified by GC–MS. Depending on the temperature and atmosphere, 25–52% of CBD was transformed into other chemical substances: Δ^9^-THC, Δ^8^-THC, cannabinol and cannabichromene were the predominant pyrolysates in both conditions, all formed by cyclization reaction. THC was the main pyrolysis product at all temperatures under both oxidative and inert conditions. Our results point out that CBD in e-cigarettes can be considered as a precursor of THC, thus it bears all the dangers related to this psychoactive compound. Our findings are fundamental contributions to the safety profile of CBD-based e-cigarettes.

## Introduction

In recent years, the consumption of *Cannabis sativa-*based products has been on the rise. The main bioactive compounds of the plant are phytocannabinoids, more than 110 of which have been identified so far^1^. (−)-*trans*-Δ^9^-Tetrahydrocannabinol (THC) and cannabidiol (CBD) are the major substances of *Cannabis*, the former known for its psychoactivity. Due to the widespread use of the plant for recreational purposes, plant breeding aimed at production of plant material rich in THC for decades. Nowadays, it seems like that the focus has been shifted from THC to CBD, since there is a growing demand from the food supplement industry for THC-free extracts with CBD as the major constituent. Cannabidiol (CBD) is a non-psychoactive compound possessing several supposed and proved beneficial health effects.

In the US, a record-breaking increase in CBD sales was generated in the past 2 years. In general, oils, tinctures, soft gels as well as liquids are found to be the most widely used forms for CBD preparations. Approximately one fifth of the US population have used some type of CBD related products in recent years^2^. Flowers, leaves, seeds from the cannabis plant are known to have traditionally been used as anti-inflammatory, pain relieving, antidepressant, and anticonvulsant remedies^3^. Authorities both in the EU and USA have lately approved the CBD-containing medicine Epidiolex^®^ for treating children of age two or older with severe epileptic seizures. However, the current rise of *Cannabis* is only partly related to the medicinal use of CBD. Most of the CBD-containing products are marketed as food supplements.

Besides medical and food use, other CBD formulations, including liquids for electronic cigarettes, have also emerged. The popularity of these liquids is increasing. In the Washington State area, cannabis extracts used for inhalation possessed 21.2% of the purchases, which was almost one and a half times higher in 2016 than it was in 2014^4^. Despite the safety claims of e-cigarettes, in some cases the appearance of lung injuries by vaping e-liquids containing CBD as an additive were reported. The possibly harmful pyrolysis products could have toxic effects in the human body^5^. Moreover, the evaporated CBD itself might increase the risk of cardiovascular events^6^.

Lawfully produced CBD products are available in the European Union but not all member states allow the distribution of them. In a French court decision, company directors from the Czech Republic were sentenced to suspended imprisonment for marketing electronic cigarette cartridges in France. Legislative authorities in France could prohibit the marketing of *Cannabis sativa* extracts based on the narcotic hallmark of CBD, thus labeling distributors as criminals^7^, even though CBD itself is not psychoactive and is not reckoned as a controlled substance by the UN Commission on Narcotic Drugs.

Smoking or using e-cigarette exposes compounds to temperatures sufficient to promote chemical reactions, modifying the composition of the smoke this way. It has been reported even in the 1970’s that CBD is partly transformed to THC in case smoking as a cigarette^8^. This was reassured later in pyrolysis studies performed at 700 °C^9^ or under simulated smoking conditions^10^, however, this pyrolysis reaction has not been studied extensively. The widespread use of CBD in e-cigarettes necessitates quantitative data on the pyrolytic transformation of CBD at lower pyrolysis temperatures. The aim of our research work presented here was to analyze the pyrolysis products of CBD by adopting standard heating conditions used in electronic cigarettes^11,12^, with special focus on THC.

## Results

Depending on e-liquid fill level, coil resistance and voltage settings, the coil temperatures of e-cigarettes ranges from 110 to 1008 °C^11,13^. Typical wetted coil temperature is 200–400 °C, extremely high temperatures (around 1000 °C) have been measured for dry coils without any e-liquid^14^. In our experiments, we studied pyrolysis in the typical operating temperature range of e-cigarettes (250–400 °C) under both inert and oxidative conditions. Additional experiments were performed at 500 °C in both atmospheres to test thermal degradation pattern at elevated temperatures.

The composition of CBD pyrolysates obtained in inert and oxidative atmospheres are summarized in Tables 1 and 2, respectively. The obtained data clearly present the thermal instability of CBD. Depending on the temperature and atmosphere, 25–52% of CBD transformed into other chemical substances during the experiments. In the absence of oxygen, 23 pyrolysis products were observed, 15 of which were identified (see Table 1). These compounds represented 81–97% of the pyrolysis products (at lower temperatures, between 250–400 °C, 93–97%). In the presence of oxygen, 22 pyrolysis products were detected (see Table 2), the identified 15 components represent the 88–96% of the degradation products in the studied temperature range. Most of the degradation products (i.e. 18 compounds) appeared among both inert and oxidative conditions. Additional four products were observed exclusively in oxidative, while five degradation products exclusively in inert atmosphere.

**Table 1:** Product distribution of CBD at various temperatures in inert atmosphere.

No.	No.^a^	Compound	t_R_ (min)	Mw (g/mol)	I 250 °C		I 300 °C		I 400 °C		I 500 °C	
					Mean (%)	SD (%)	Mean (%)	SD (%)	Mean (%)	SD (%)	Mean (%)	SD (%)
1	2	*p*-Mentha-1,3,8-triene	12.88	134	n.d		n.d		1.08	0.35	n.d	
2	3	*p*-Mentha-1,5,8-triene	13.23	134	n.d		n.d		1.34	0.05	1.70	0.36
3	4	*p*-Cymene	13.31	134	n.d		n.d		n.d		1.93	0.13
4	5	*p*-Cymenene	14.78	132	n.d		n.d		n.d		0.48	0.14
5	6	*p*-Mentha-1,4,8-triene	14.88	134	n.d		n.d		n.d		1.83	0.48
6	8	Olivetol	26.43	180	n.d		n.d		0.50	0.19	2.25	0.45
7	9	2,2-Dimethyl-7-pentyl-2*H*-chromen-5-ol	27.21	246	n.d		n.d		n.d		1.00	0.51
8	10	Cannabicyclol	29.74	314	0.12	0.02	n.d		0.29	0.11	0.38	0.01
9	11	Unidentified	29.83	314	0.15	0.04	n.d		0.12	0.02	0.22	0.08
10	12	Unidentified A^b^	29.98	312	0.14	0.03	n.d		0.30	0.08	1.97	0.25
11	13	Unidentified	30.08	314	n.d		n.d		n.d		0.59	0.06
12	14	6-Methyl-3-pentyl-9-(propan-2-ylidene)-5a,6,7,8,9,9a-hexahydro-dibenzo[*b*,*d*]furan-1-ol	30.28	314	0.39	0.06	0.15	0.03	0.33	0.12	0.75	0.07
13	15	Unidentified	30.45	312	n.d		n.d		n.d		0.44	0.03
14	16	5-Pentyl-2-(4,6,6-trimethylbicyclo[3.1.1]hept-3-en-2-yl)benzene-1,3-diol	30.51	314	0.50	0.04	n.d		0.40	0.23	1.42	0.11
15	18	Unidentified B^b^	30.62	312	n.d		0.18	0.07	0.33	0.17	0.32	0.07
16	19	Cannabichromene	30.73	314	7.00	0.49	3.17	0.94	5.64	1.76	2.35	0.54
17	20	CBD	30.82	314	48.20	5.13	74.62	4.40	62.11	9.53	67.66	5.51
18	21	Unidentified C^b^	31.10	314	0.56	0.06	0.62	0.18	0.70	0.12	1.07	0.16
19	22	Unidentified D^b^	31.17	314	0.45	0.02	n.d		0.46	0.10	0.88	0.22
20	23	Δ^8^-THC	31.25	314	4.94	0.67	1.64	0.32	2.73	0.68	2.76	0.63
21	24	Δ^6a,10a^-THC	31.39	314	n.d		n.d		n.d		1.36	0.29
22	25	Δ^9^-THC	31.49	314	33.94	4.07	17.95	2.96	20.17	4.42	5.88	1.57
23	27	Unidentified E^b^	31.82	314	n.d		n.d		n.d		0.53	0.07
24	28	Cannabinol	32.41	310	3.64	0.07	1.66	0.33	3.49	1.49	2.25	0.21

Percentages are calculated using peak areas of the total ion current chromatograms.

*n.d.* below detection limit, *t*_*R*_ retention time, *M*_*W*_ molecular weight.

^a^Compound ID number.

^b^Unidentified compounds which appear in both inert and oxidative conditions.

**Table 2:** Product distribution of CBD at various temperatures in oxidative atmosphere.

No	No.^a^	Compound	t_R_ (min)	Mw (g/mol)	O 250 °C		O 300 °C		O 400 °C		O 500 °C	
					Mean (%)	SD (%)	Mean (%)	SD (%)	Mean (%)	SD (%)	Mean (%)	SD (%)
1	1	Unidentified monoterpene	11.62	134	n.d		n.d		0.42	0.07	n.d	
2	2	*p*-Mentha-1,3,8-triene	12.76	134	0.94	0.15	1.37	0.65	0.64	0.18	n.d	
3	3	*p*-Mentha-1,5,8-triene	13.17	134	1.00	0.04	1.16	0.52	1.70	0.07	3.64	0.62
4	4	*p*-Cymene	13.32	134	n.d		n.d		n.d		2.87	0.45
5	5	*p*-Cymenene	14.83	132	n.d		n.d		0.47	0.19	1.61	0.69
6	6	*p*-Mentha-1,4,8-triene	14.92	134	n.d		n.d		1.30	0.41	3.60	1.09
7	7	6-Pentylbenzofuran-4-ol	25.99	204	n.d		0.28	0.11	n.d		n.d	
8	8	Olivetol	26.46	180	n.d		0.21	0.07	0.76	0.21	n.d	
9	10	Cannabicyclol	29.74	314	1.08	0.27	0.21	0.07	0.64	0.13	0.44	0.10
10	12	Unidentified A^b^	29.98	312	0.30	0.04	0.41	0.03	1.02	0.29	0.33	0.11
11	14	6-Methyl-3-pentyl-9-(propan-2-ylidene)-5a,6,7,8,9,9a-hexahydro-dibenzo[*b*,*d*]furan-1-ol	30.28	314	0.23	0.05	0.34	0.04	0.68	0.17	n.d	
12	16	5-Pentyl-2-(4,6,6-trimethylbicyclo[3.1.1]hept-3-en-2-yl)benzene-1,3-diol	30.51	314	0.11	0.02	n.d		0.72	0.42	n.d	
13	17	Unidentified	30.54	312	0.19	0.04	0.78	0.10	1.75	2.40	n.d	
14	18	Unidentified B^b^	30.61	312	0.26	0.05	0.58	0.16	0.19	0.05	n.d	
15	19	Cannabichromene	30.73	314	5.03	0.95	5.74	0.59	5.32	0.67	2.36	1.05
16	20	CBD	30.81	314	62.02	5.09	61.58	2.95	52.19	6.56	64.41	9.98
17	21	Unidentified C^b^	31.10	314	0.74	0.13	1.04	0.14	1.27	0.29	n.d	
18	22	Unidentified D^b^	31.15	314	n.d		0.52	0.07	0.72	0.18	n.d	
19	23	Δ^8^-THC	31.25	314	3.57	0.74	2.54	0.23	3.38	0.73	2.10	0.66
20	25	Δ^9^-THC	31.48	314	16.09	3.96	12.85	0.86	13.45	2.38	8.55	3.77
21	26	Cannabielsoin	31.50	330	3.44	0.14	3.83	0.50	2.56	0.27	2.89	0.68
22	27	Unidentified E^b^	31.81	314	n.d		n.d		0.39	0.02	1.63	0.56
23	28	Cannabinol	32.41	310	5.00	0.34	6.56	0.49	10.44	0.82	5.59	2.55

Percentages are calculated using peak areas of the total ion current chromatograms.

*n.d.* below detection limit, *t*_*R*_ retention time, *M*_*W*_ molecular weight.

^a^Compound ID number.

^b^Unidentified compounds which appear in both inert and oxidative conditions.

**Figure 1:**
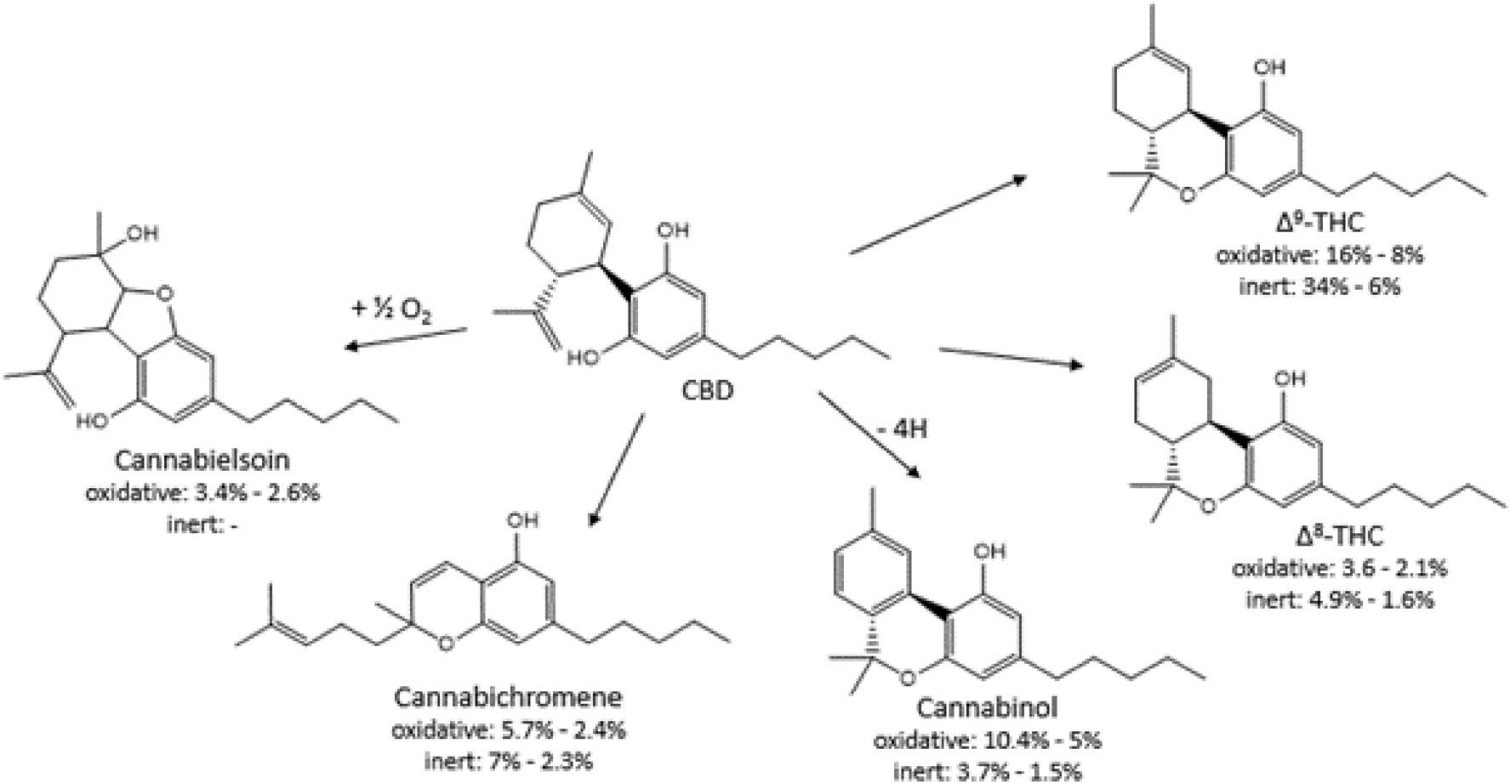
The major thermal decomposition routes of CBD in inert (helium) and oxidative (9% oxygen in nitrogen) atmosphere in a temperature range of 250–500 °C.

The four most intense products, namely Δ^9^-THC, Δ^8^-THC, cannabichromene and cannabinol account for more than 95% of the decomposition products at 250 and 300 °C pyrolysis temperatures in inert atmosphere. Under oxidative conditions an additional product, cannabielsoin appeared. The ratio of the aforementioned five decomposition products is more than 80% under oxidative conditions up to 300 °C. All of these compounds formed by cyclization reaction (see Fig. 1). Cyclization of phenolic flavors to bicyclic compounds under simulated tobacco heating conditions at 300 °C was reported earlier^15^. The phenolic O of thymol or ethylvanillin link to a geometrically favorable position of a sterically adjacent side group of the molecule, forming a bicyclic compound. Analogously, in the present case one of the phenolic O of CBD linked to the tertiary carbon of the isopropenyl group, thus forming a sterically favored six-membered ring and resulting Δ^9^-THC molecule. However, Δ^9^-THC formed also under inert atmosphere. The related cyclization can be formally described as an intramolecular Markovnikov addition of the phenolic OH onto the double bond of the isopropenyl group. Thus, no oxidation step is needed by the formation of Δ^9^-THC. The psychoactive Δ^9^-THC was the main detected compound, accounting up to 42% and 70% of the decomposition products under oxidative and inert conditions, respectively, at all applied temperatures. One possible reason of the lower Δ^9^-THC amount in oxidative atmosphere measured in our study could be the higher decomposition rate of the formed Δ^9^-THC in the oxidative ambient. Increased degradation rate of Δ^9^-THC was published in cannabis resin sample exposed to air comparing to that of stored in a sealed plastic bag at ambient temperature^16^ indicating the role of oxygen in Δ^9^-THC decomposition.

Among THC isomers, Δ^8^-THC has also psychotropic effects according to the recent review of the Expert Committee on Drug Dependence of the World Health Organization (WHO)^17^. The Δ^8^-THC molecule formed by an additional isomerization to the cyclization during thermal treatment. Both Δ^9^-THC and Δ^8^-THC were formed at higher rate in inert atmosphere and at relative lower temperatures (250–300 °C). By increasing the temperature, the relative yield of THC decreased, while other decomposition reactions became more pronounced.

According to earlier studies, cannabinol derives by cyclization, dehydration, and aromatization of CBD, probably through a THC intermediate during long-term storage^16^*.* Notable amount of cannabinol was detected in the present study as a thermal degradation product of CBD. The relative amount of cannabinol was significantly higher in oxidative conditions at each studied temperature, indicating the effect of oxygen in the reaction mechanism. In addition to the driving force of the aromatization in both atmospheres, dehydrogenation by oxygen with water elimination may play additional role in oxidative atmosphere. The most intense cannabinol formation (10.4%) was observed at 400 °C in oxidative atmosphere.

Cannabichromene also formed through cyclization reaction. However, one of the phenolic O of the CBD molecule attacked the double bond of the cyclohexene ring at C substituted with methyl group in this case, while the cyclohexene ring opened up by C–C scission to form the chromene frame. Formation of cannabichromene was more pronounced in inert atmosphere compared to the oxidative condition at 250 °C. At higher temperatures there was no significant difference and the relative yield of cannabichromene was decreased.

Cannabielsoin was only detected under oxidative conditions. In this transformation, one of the phenolic O of CBD linked similarly to the double bond of its cyclohexene ring, but at the secondary carbon, while a hydroxyl group formed at the adjacent C substituted with methyl group via the oxidative medium. The relative intensity of cannabielsoin was around 3%, and its quantity was not much affected in the temperature range of 250–500 °C.

At higher temperatures, the share of the decomposition products formed through cyclization reaction was decreased, while the relative intensity of smaller molecules formed by C–C bond scission increased in the pyrolyzate. These identified products were formed by the scission of the bond connecting the pentylbenzenediol and the *p*-mentha-1,8-dienyl moieties of the CBD molecule. Menthatriene isomers appeared at 250 °C in oxidative atmosphere, while in inert atmosphere only above 400 °C, indicating that the presence of oxygen promoted the cleavage of the molecule. The relative amount of menthatrienes were the highest at 500 °C in both inert and oxidative atmospheres.

## Discussion

Here we present the first extensive analysis of the pyrolysis of CBD under conditions relevant in case of using the substance in e-cigarettes. THC was the main pyrolysis product at all temperatures under both oxidative and inert conditions. The typical THC content (the integer number closest to the median values) of joints used for recreational purposes is around 7 mg^18^. A Swiss analysis based on 20 samples found that the total CBD content of e-liquids ranged between 0.182 and 3.346% and differed in half of the samples from the claimed content^19^. According to a study, two e-liquids were labeled to contain 3.3 mg/mL of CBD, however they were found to contain 6.5 and 7.6 mg/mL of CBD^20^. On the basis of our results, it might be assumed that up to 0.5–1 mg of THC may be intaken by vaping each mL of CBD-containing e-liquid. However, it should be mentioned that the applied experimental method revealed the thermal degradation of pure CBD. Therefore, the possible effect of the vaping fluid (e.g. PG/VG^20^) on the stability and breakdown pattern was not taken into consideration. Nevertheless, due to the observed high conversion rate of CBD to THC, the results highly draw attention on the risk of CBD-containing e-liquids. The heating time in an e-cigarette may vary depending on its type and customer habit. Despite the relatively long isothermal period (5 min) applied in the pyrolysis method, a short residence time in the hot zone of the pyrolyzer is expected, because the boiling point of CBD and its degradation products is relatively low (155–185 °C) and high flow rate (276 mL/min) was applied during pyrolysis.

Recently, the incidence of the so-called vaping-associated pulmonary illness (VAPI) has been increasing (this condition is also known as EVALI, e-cigarette or vaping product use-associated lung injury). Until February 2020, more than 2800 patients had been hospitalized in the US due to VAPI, with a total of 68 deaths reported ^21^. Although the etiology of VAPI has not been elucidated yet, nicotine, THC, CBD, and vitamin E are among the most suspected ingredients with a link to the pathogenesis of the disease ^22^.

The presence of CBD in e-cigarettes might pose dual risk. First, as a precursor of the psychoactive THC, it bears all the dangers related to THC use. Consumers of CBD-containing e-cigarettes may also be involuntarily exposed to the psychoactive THC. Moreover, its use in e-cigarette liquids is not regulated; therefore, the exposure of e-cigarette users to THC is unknown. And second, CBD itself is a pharmacologically active compound with remarkable activities on the central nervous and the cardiovascular system and is marketed as prescription-only medicine with several potential adverse effects such as somnolence, decreased appetite, diarrhea, pyrexia, fatigue, and vomiting^23^.

The results presented here might be important contributions to the legal discretion of CBD. Although according to the French law, the distribution of CBD-containing e-cigarette cartridges was considered as infringements of the legislation on poisonous substances, the Court of Justice of the European Union had a different interpretation^24^. Albeit CBD itself is void of psychoactivity, our results underline, that remarkable amount of THC might be formed from CBD during evaporation in an e-cigarette. Therefore, the standpoint, that CBD does not appear to have any psychotropic effect or any harmful effect on human health on the basis of available scientific data, should be reconsidered. From a scientific point of view, when used in an e-cigarette, CBD is the precursor of THC, although this fact is not reflected yet in drug policy and legislation.

## Methods

A 1 mg/mL CBD in methanol solution was purchased from Supelco (certified reference material, Cerilliant), and kept at − 20 °C till the analysis. The experiments were performed either in a gas mixture of 9.34% (*n*/*n*) oxygen and 90.66% (*n*/*n*) nitrogen or in helium atmosphere.

The experimental method to simulate the low-temperature tobacco heating conditions was developed in our earlier study^12^. This method was adopted and modified by using various temperatures^11^ to study the breakdown pattern of CBD; therefore, the experimental conditions are described here briefly.

Py-GC/MS analyses have been carried out using a Pyroprobe 2000 (CDS Analytical, Oxford, PA, USA) pyrolyzer equipped with a platinum heating coil and a quartz sample tube. A total 15 µL aliquot of solution was dispensed in 5 µL portions onto a piece of quartz wool placed in the quartz tube and it was rested for 3 min at room temperature to allow evaporating the majority of the solvent after each portion. The quartz tube was placed in the Pyroprobe, at room temperature, which was then inserted into the pre-heated pyrolysis chamber. The temperature of the pyrolysis chamber was 280 °C, except in case of pyrolysis experiments performed at 250 °C, when the chamber temperature was set at 250 °C as well. The pyrolysis chamber was flushed at a 276 mL/min flow rate by the applied gas mixture. The sample was then heated at a maximal heating rate (set at 999 °C/s) to the final pyrolysis temperature. The experiments were performed at five different pyrolysis temperatures in a temperature range of 250–500 °C, using 5 min isotherm period in the case of each pyrolysis temperature. Oxidative experiments were performed in a gas mixture of 9% oxygen and 91% nitrogen. In order to reveal the role of oxygen in the thermal degradation reactions, additional experiments were performed in helium atmosphere, applying the same pyrolysis temperatures. The volatile products were purged on-line to a DB-1701 capillary column (30 m × 0.25 mm i.d., 0.25 µm film thickness) of the GC/MS (Agilent 6890 GC/5973 MSD) system. At the end of the pyrolysis, the pyrolysis gas flow was closed, and helium carrier gas was supplied to the GC/MS. Solvent delay of 7 min was applied to protect the MS. The GC oven was programmed to 7 min isotherm period at 40 °C before increase to 280 °C at a rate of 10 °C/min. The range of *m*/*z* 29–400 was scanned by the mass spectrometer in EI mode (70 eV). At least three parallel experiments were performed at each temperature. The identification of the pyrolysis products (see Supplementary Table S1) was based on the combined Wiley Registry 9th edition/NIST 2011 mass spectral library and literature data^25–29^. The percentages of the compounds were estimated using the peak areas of the total ion current chromatograms.

## Supplementary Information

Supplementary Information.
