# Supplementary material for: CBD, a precursor of THC in e-cigarettes
Source: Sci Rep. 2021 Apr 26;11:8951. doi: 10.1038/s41598-021-88389-z (PMC8076212; doi:10.1038/s41598-021-88389-z)
Supplement: Supplementary file 1 — Supplementary Information. [file 41598_2021_88389_MOESM1_ESM.pdf]

Supporting information to

CBD, a precursor of THC in e-cigarettes

Zsuzsanna Czégény,[1] Gréta Nagy,[1] Bence Babinszki,[1] Ákos Bajtel,[2] Zoltán Sebestyén, [1] Tivadar Kiss,[2] Boglárka Csupor-Löffler,[3] Barbara Tóth,[2] Dezső Csupor[2,3,*]

[1] Institute of Materials and Environmental Chemistry, Research Centre for Natural Sciences, Eötvös Loránd Research Network, 1117 Budapest, Hungary

[2] Department of Pharmacognosy, Faculty of Pharmacy, University of Szeged, 6720 Szeged, Hungary

[3] Institute of Translational Medicine, Medical School, University of Pécs, 7624 Pécs, Hungary

*Corresponding author:

Dr. Dezső Csupor, email: csupor.dezso@pharmacognosy.hu; Tel: +36 62 545 559

Table S1. EI mass spectra of the detected CBD decomposition products. Library mass spectra for comparison are also indicated in the cases of the identified components.

#	Compound	t_R (min)	Mw (g/mol)	EI mass spectra
1	Unidentified monoterpene	11.62	134	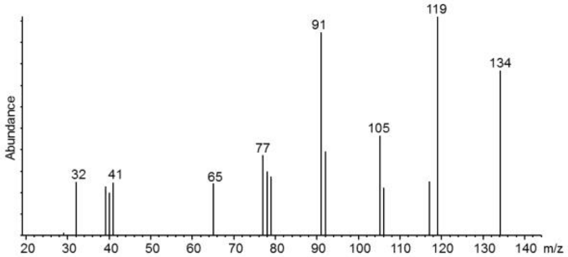
2	*p*-Mentha-1,3,8-triene 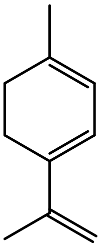	12.76	134	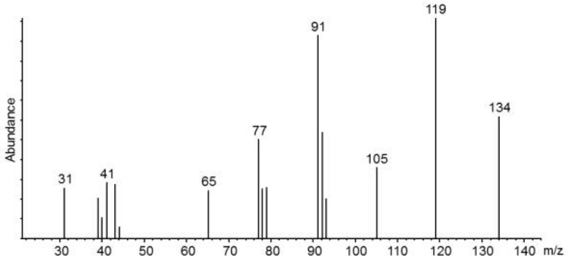

#	Compound	t_R (min)	Mw (g/mol)	EI mass spectra
3	*p*-Mentha-1,5,8-triene 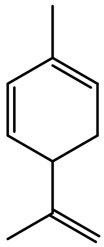	13.17	134	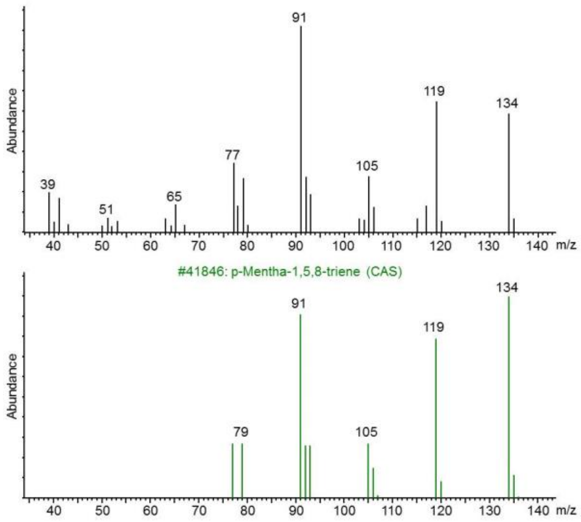
4	*p*-Cymene 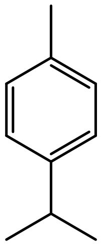	13.32	134	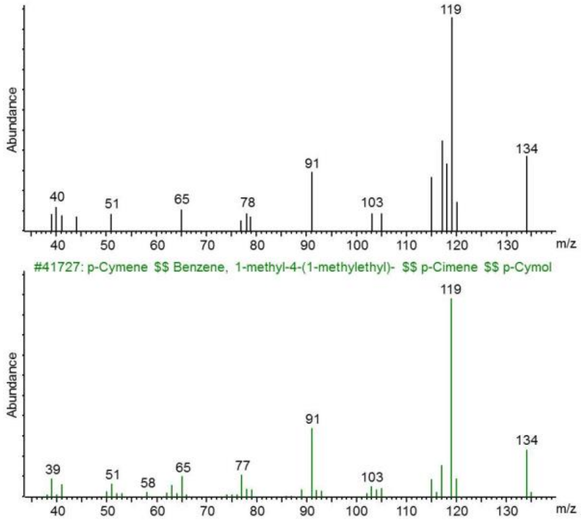
5	*p*-Cymenene 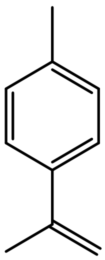	14.83	132	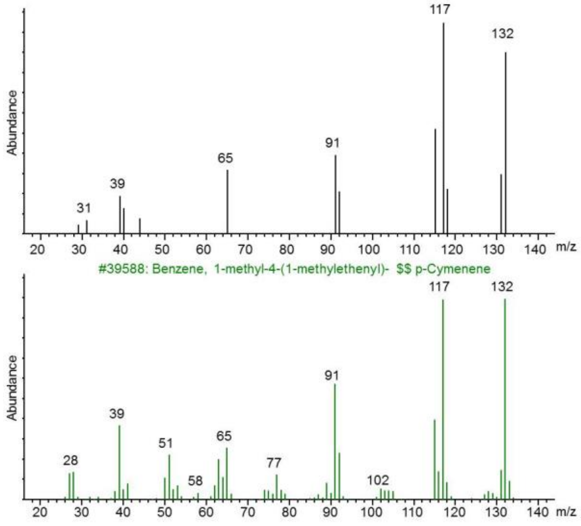

#	Compound	t_R (min)	Mw (g/mol)	EI mass spectra
6	*p*-Mentha-1,4,8-triene<br>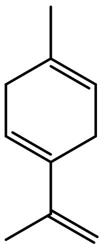	14.92	134	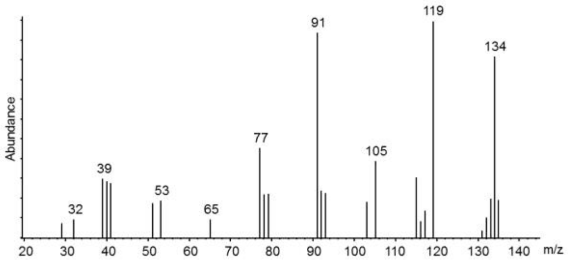
7	6-Pentylbenzofuran-4-ol<br>(identification based on Ref.25 and Ref.29)<br>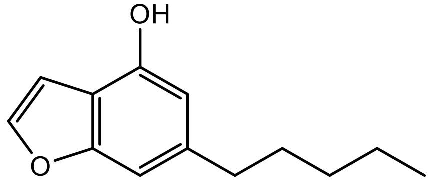	25.99	204	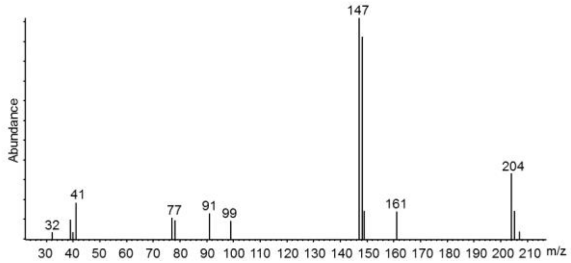
8	Olivetol<br>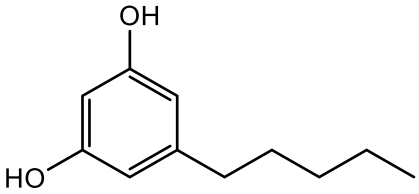	26.43	180	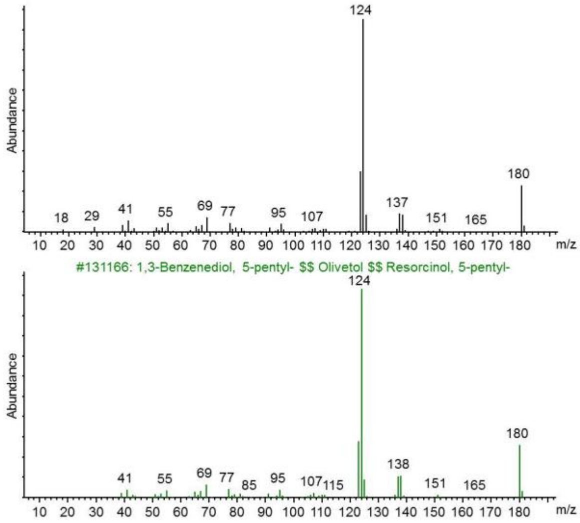
9	2,2-Dimethyl-7-pentyl-2*H*-chromen-5-ol<br>(identification based on Ref.25)<br>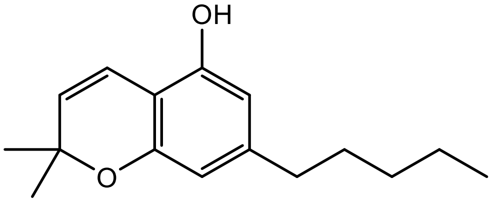	27.21	246	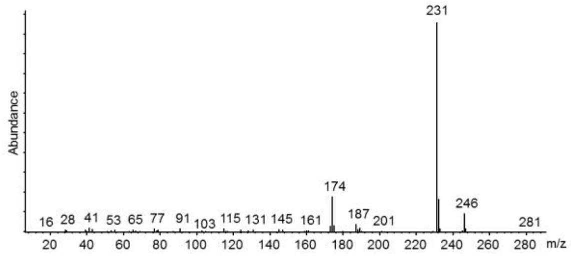
10	Cannabicyclol<br>(identification based on Ref.26)<br>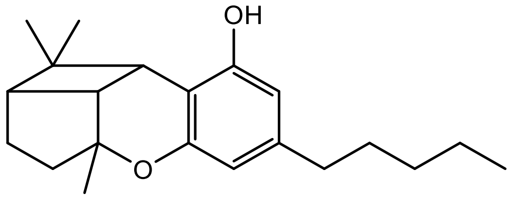	29.74	314	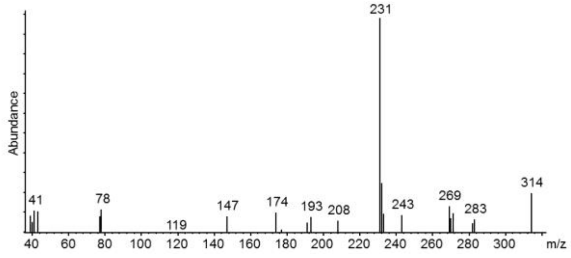

#	Compound	t_R (min)	Mw (g/mol)	EI mass spectra
11	Unidentified	29.83	314	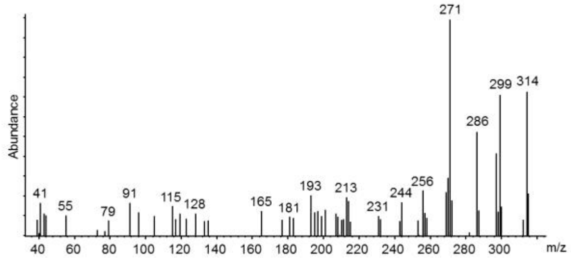
12	Unidentified A	29.98	312	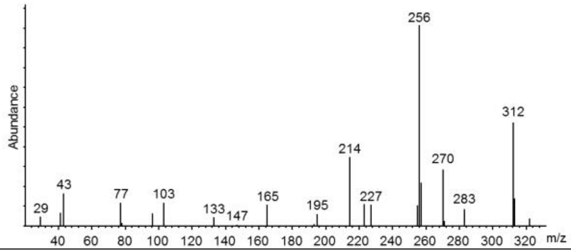
13	Unidentified	30.08	314	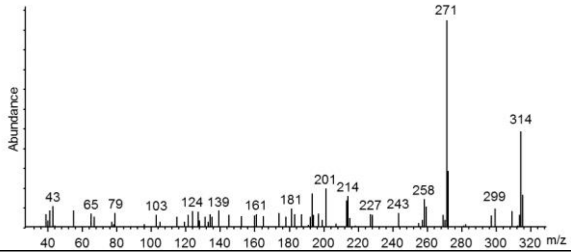
14	6-Methyl-3-pentyl-9-(propan-2-ylidene)-5a,6,7,8,9,9a-hexahydrodibenzo[b,d]-furan-1-ol (identification based on Ref. 27) 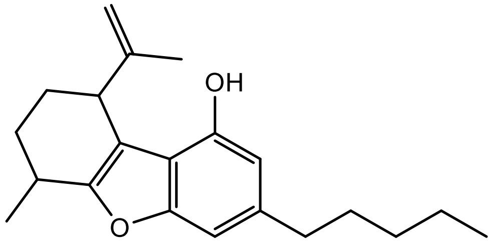	30.28	314	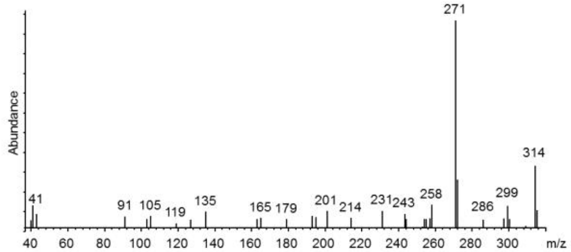
15	Unidentified	30.45	312	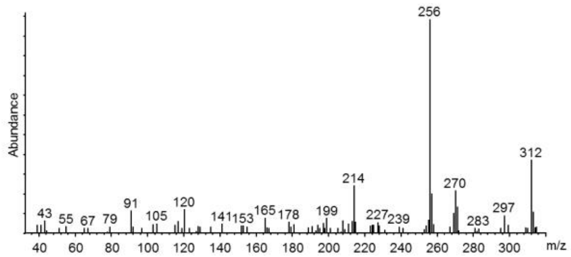

#	Compound	t_R (min)	Mw (g/mol)	EI mass spectra
16	5-Pentyl-2-(4,6,6-trimethylbicyclo[3.1.1]hept-3-en-2-yl)benzene-1,3-diol 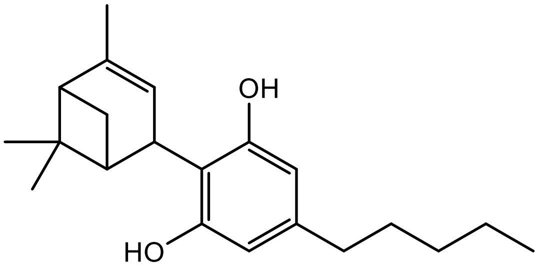	30.51	314	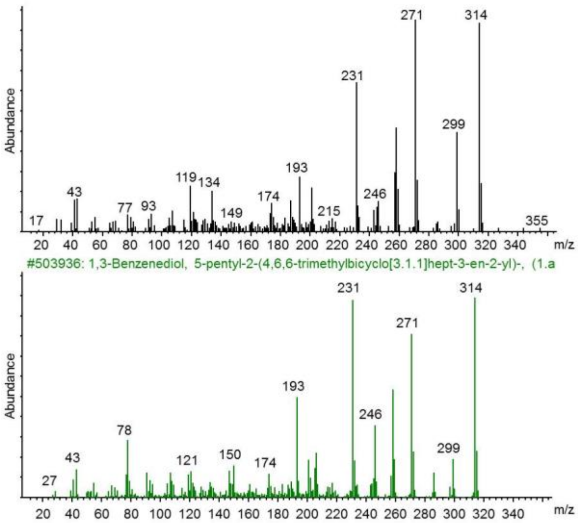
17	Unidentified	30.54	312	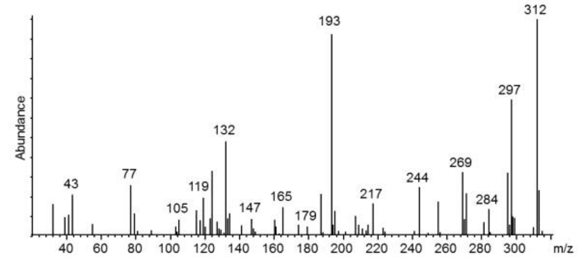
18	Unidentified B	30.61	312	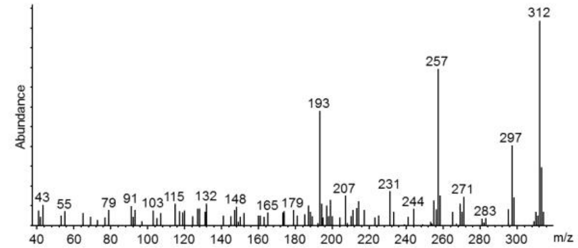
19	Cannabichromene 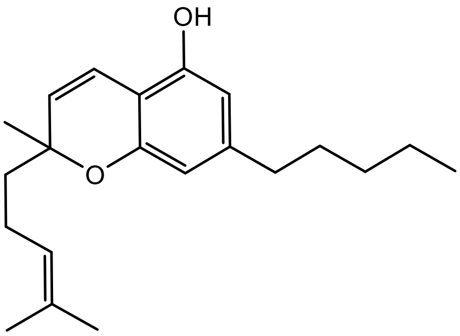	30.73	314	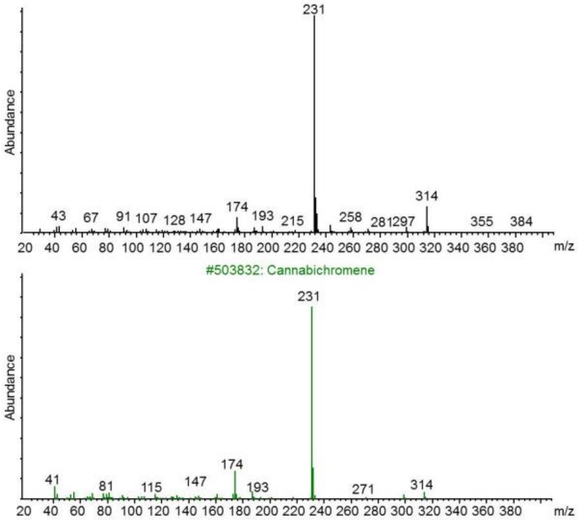

#	Compound	t_R (min)	Mw (g/mol)	EI mass spectra
20	CBD 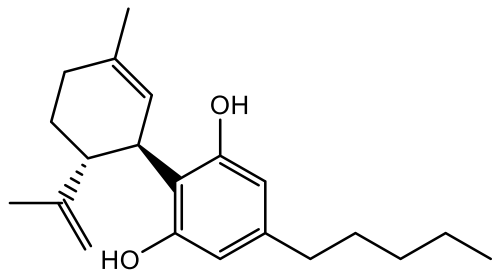	30.81	314	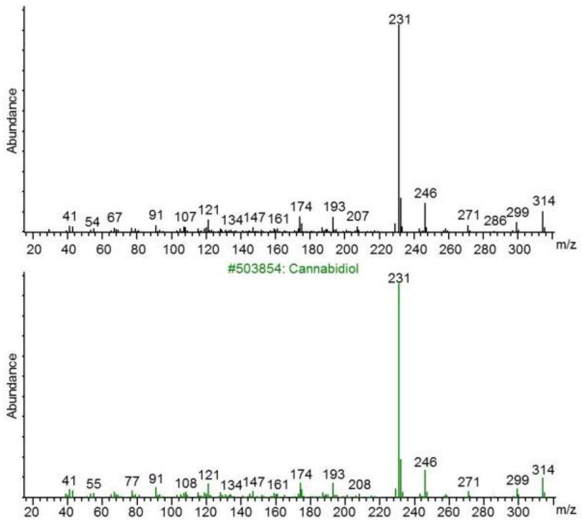
21	Unidentified C	31.10	314	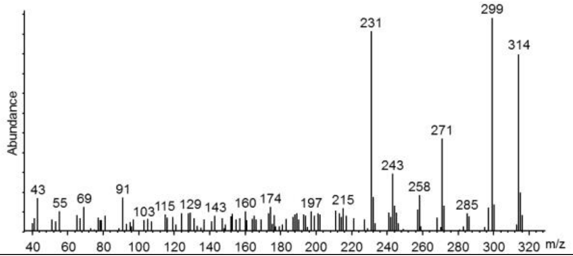
22	Unidentified D	31.15	314	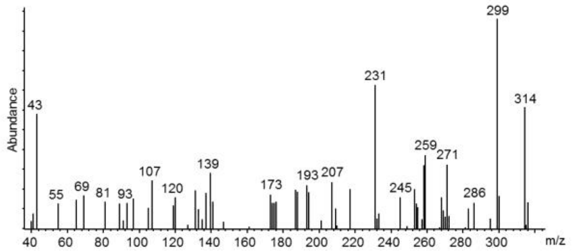
23	Δ^8-THC 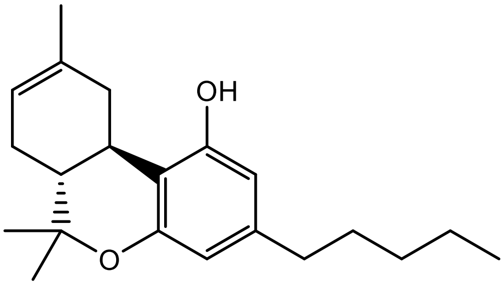	31.25	314	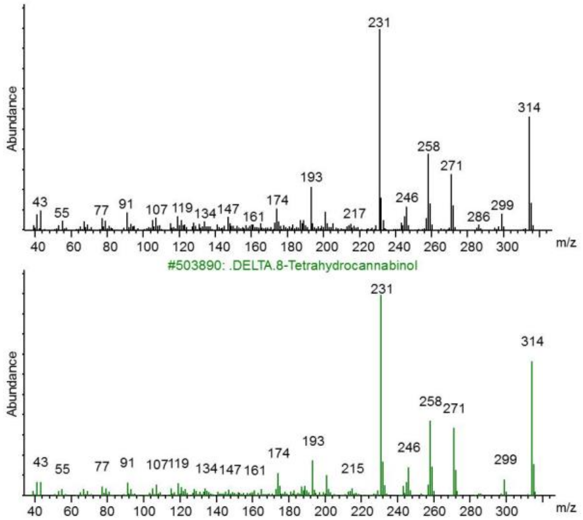

#	Compound	t_R (min)	Mw (g/mol)	EI mass spectra
24	$\Delta^{6a,10a}$-THC (identification based on Ref. 28) 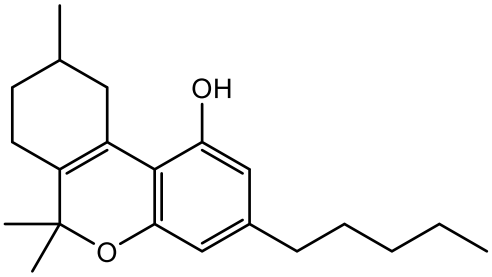	31.39	314	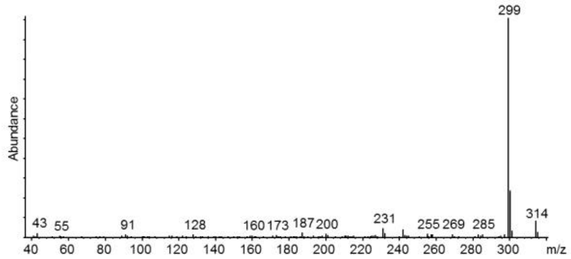
25	Δ^9-THC 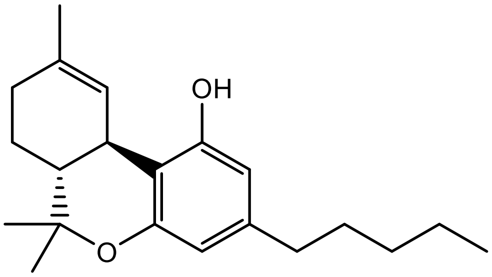	31.48	314	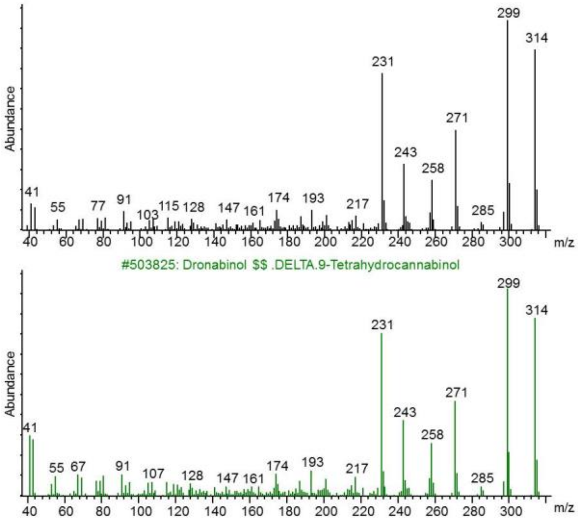
26	Cannabielsoin 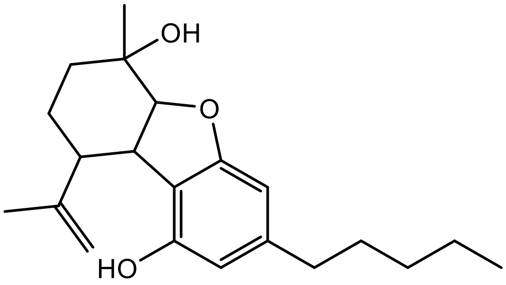	31.50	330	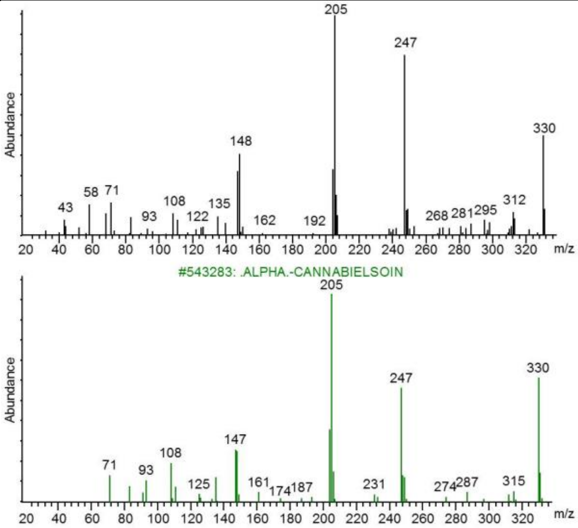
27	Unidentified E*	31.81	314	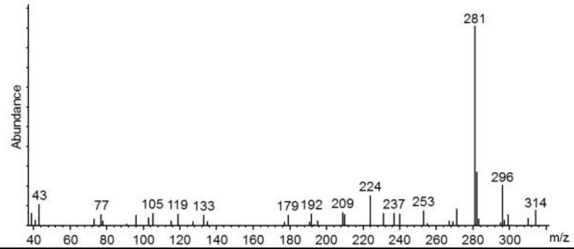

#	Compound	t_R (min)	Mw (g/mol)	EI mass spectra
28	Cannabinol 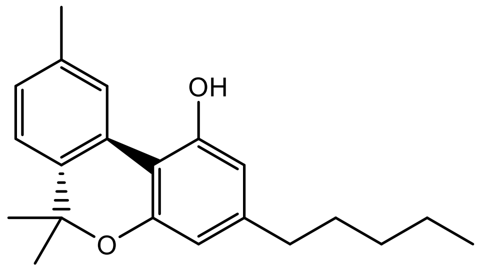	32.41	310	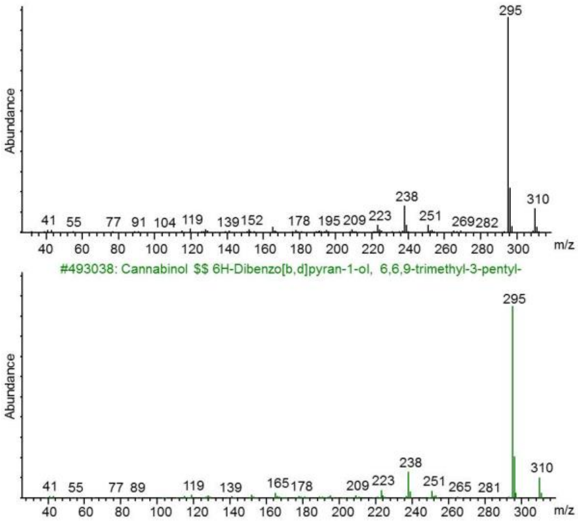
